# Pathogenesis and treatment of cytokine storm in COVID-19

**DOI:** 10.3906/biy-2105-37

**Published:** 2021-08-30

**Authors:** Mehmet SOY, Gökhan KESER, Pamir ATAGÜNDÜZ

**Affiliations:** 1 Division of Rheumatology, Department of Internal Medicine, Faculty of Medicine, Altınbaş University, Bahçelievler Medical Park Hospital, İstanbul Turkey; 2 Division of Rheumatology, Department of Internal Medicine, Faculty of Medicine, Ege University, İzmir Turkey; 3 Division of Rheumatology, Department of Internal Medicine, Faculty of Medicine, Marmara University, İstanbul Turkey

**Keywords:** COVID-19, SARS-CoV-2, cytokine storm, acute respiratory distress syndrome, tocilizumab, anakinra

## Abstract

COVID-19 is a viral infection caused by the severe acute respiratory syndrome coronavirus 2 (SARS-CoV-2) that killed a large number of patients around the world. A hyperinflammatory state resulting in a cytokine storm and adult respiratory distress syndrome seems to be the major cause of the death. Many mechanisms have been suggested in the pathogenesis of COVID-19 associated cytokine storm (COVID-CS). Insufficient viral clearance and persistence of a strong cytokine response despite inadequate antiviral immunity seem to be the main mechanisms underlying the pathogenesis. The diagnosis of COVID-19 is based on relatively constant clinical symptoms, clinical findings, laboratory tests, and imaging techniques, while the diagnosis of COVID-CS is a rather dynamic process, based on evolving or newly emerging findings during the clinical course. Management of COVID-19 consists of using antiviral agents to inhibit SARS-CoV-2 replication and treating potential complications including the cytokine storm together with general supportive measures. COVID-CS may be treated using appropriate immunosuppressive and immunomodulatory drugs that reduce the level of inappropriate systemic inflammation, which has the potential to cause organ damage. Currently corticosteroids, IL-6 blockers, or IL-1 blockers are most widely used for treating COVID-CS.

## 1. Introduction and historical background

Coronavirus disease 2019 (COVID-19) is a severe acute respiratory syndrome that emerged in China in December 2019. After its rapid spread worldwide, the World Health Organization (WHO) declared the situation as a pandemic on March 11, 2020. It is a clinical syndrome caused by a new coronavirus, called severe acute respiratory syndrome coronavirus 2 (SARS-CoV-2), which is a beta-coronavirus similar to two other coronaviruses that have caused fatal infections in the last two decades. The other two coronaviruses, namely the severe acute respiratory syndrome coronavirus (SARS-CoV) and the Middle East respiratory syndrome coronavirus (MERS-CoV) were responsible for fatal outcomes (Paules et al., 2020). SARS-CoV-2 shows 79.5% and 50% genome-wide sequence identity with SARS-CoV and MERS-CoV, respectively. However, the SARS-CoV-2 spike protein exhibits a 10–20 times higher affinity for the cellular angiotensin-converting enzyme-2 (ACE-2) receptors in humans (Wrapp et al., 2020).

Coronaviruses (CoVs) are large, enveloped, RNA viruses that can be divided into four types: alpha, beta, delta, and gamma. Of these, alpha and beta CoVs are known to infect humans and are called HCoV. The surface spike (S) glycoprotein of coronaviruses is critical for binding to host cell receptors. The emergence of SARS-CoV and MERS-CoV followed by this third, highly pathogenic zoonotic HCoV, illustrates the threat posed by this viral family (Ganesh et al., 2016; Su et al., 2016; Paules et al., 2020). However, SARS-CoV and MERS-CoV have lost their effectiveness within time (Paules et al., 2020). Unfortunately, the SARS-COV-2 virus continues to spread and causes mortality and morbidity in humans without losing its virulance, with more than 160 million confirmed infected cases and more than 3 million deaths, reported to date. Until now over one billion people were vaccinated with the recently developed vaccines against the virus to.^1^World Health Organization (WHO) (2021). Name of resource [online]. Website https://www.who.int/ [accessed 00 Month Year].

SARS-CoV-2 remains asymptomatic or causes mild symptoms in most patients and is less lethal than MERS-CoV. However, up to 10%–20% of cases (usually older people with comorbidities) may develop interstitial pneumonia acute respiratory distress or septic shock, which can progress to acute respiratory distress syndrome (ARDS). In these cases, a serious disease picture is characterized by a rapid deterioration of the patient’s clinical condition and the development of the features of hemophagocytic syndrome or macrophage activation syndrome (MAS). In these patients, uncontrolled and high secretion of proinflammatory cytokines, very high levels of serum ferritin and CRP levels, hepatic dysfunction, disseminated intravascular coagulation (DIC), and hypercoagulopathy is detected (Huang et al., 2020). This hyperinflammatory condition is similar to hemophagocytic lymphohistiocytosis (HLH), which was initially called secondary HLH or MAS, and also resembles the classically known chimeric antigen receptor T cell-(CAR-T) induced cytokine release syndrome. However, COVID-19 induced hyperinflammatory state incorporates some differences. For example, high serum levels of cytokines and ferritin seen in the course of COVID-19 are milder, splenomegaly and lymphadenopathy are not present, and even splenic atrophy can be detected in some COVID-19 cases (Yao et al., 2020). For these reasons, this hyperinflammatory condition may be called a cytokine storm secondary to COVID-19 or COVID-19 associated cytokine storm (COVID-CS) (Samudrala et al., 2020; Soy et al., 2020; Caricchio et al., 2021; Nigrovic et al., 2021). Since it not possible to use the diagnostic criteria for the diagnosis of classic HLH and MAS for COVID-CS, Caricchio et al. (2021) from Temple University put forward new criteria to overcome this confusion and to make an early diagnosis of COVID-CS. This severe hyperinflammation and its complications can lead to the death of the patient, if not treated swiftly, and a dynamic approach in adapting the therapy to the emerging complications is required.

Understanding the underlying pathogenetic mechanisms of COVID-19 and recognizing the broad disease spectrum ranging from mild to severe are fundamental requirements for an effective treatment strategy in critically ill patients. In this respect, it is necessary to review briefly the pathogenesis of SARS-CoV-2 infection and COVID-CS, and the underlying mechanisms.

In this article, the immunopathogenesis of COVID-19 disease, immune modalities to control the virus, and mechanisms and management of the hyperinflammation that occurs during the disease course will be discussed.

## 2. Immunopathogenesis of COVID-19

The novel coronavirus, SARS-CoV-2 is the last member of coronaviridae that can infect humans. It has four main structural proteins including the spike (S), membrane, envelope, and nucleocapsid proteins. SARS-CoV-2 carries a larger genome than any other virus known so far (26.4–31.7 kb) (Mousavizadeh et al., 2020). The S protein, which gives the typical shape of the virus, contains a special binding site that allows the virus to bind to the host cell. In the host cell, the target of the virus is the ACE-2 receptor present in many organs and systems including the heart, lungs, kidneys, and gastrointestinal tract (Chen et al., 2020). The ultrastructural examination of the viral S protein of SARS-CoV-2 revealed its similarity to the S-protein of the other CoVs. S protein contains an S1 domain capable of receptor binding and an S2 domain attached to the membrane. The S1 domain is divided into the N and C terminal domains. In SARS-CoV and SARS-CoV-2 viruses, the C terminal domain is responsible for the binding to the ACE-2 receptor of the host. But, SARS-CoV-2 has a 10- to 20-fold increased binding affinity to ACE-2 (Bourgonje et al., 2020). This binding and the following S-protein priming by the host cell protease trans-membrane protease/serine (TMPRSS) and cathepsins in the absence of TMPRSS permit the entry into the host cell (Sun et al., 2020). After the virus enters the cell, the viral RNA genome is released into the cytoplasm of the host cell and translated into structural proteins, after which the viral genome begins to be replicated and the whole virus is secreted into the cytoplasm in a vesicle. These virus-containing vesicles fuse with the plasma membrane and release the viruses, and as the host cell disintegrates the virus becomes attached to other cells. (Sun et al., 2020). 

In humans, the ACE receptor is most commonly found on the surface of the respiratory tract cells, particularly on type-2 pneumocytes. The stimulation of this receptor by the S1 domain of the SARS-CoV-2 virus downregulates ACE-2 which leads to the compensatory production of high amounts of angiotensin-2 by ACE-1 activity and causes increased permeability in the lungs. In addition, a high amount of proinflammatory cytokines and chemokines are secreted from dendritic cells and macrophages that are activated as a result of excessive and ongoing stimulation of the immune system, as described more in detail in the following paragraphs. This situation, if untreated effectively, leads to more pronounced organ damage and a hyperinflammatory state that is called COVID-CS, and can lead to the death of the patient (Samudrala et al., 2020; Soy et al., 2020).

## 3. Why cytokine storm develops in some patients with COVID-19?

**Figure 1:**
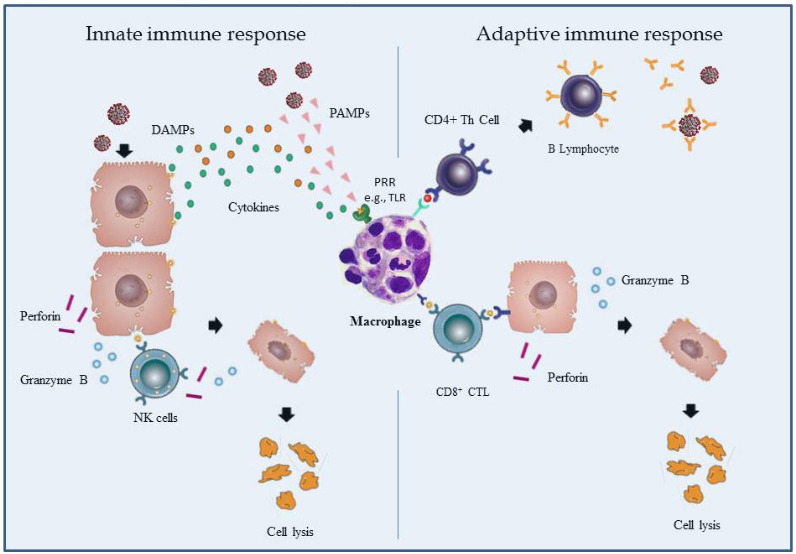
Immune response to viral pathogen.

In a normal-working immune system, when pathogens, including viruses, enter the body, their antigens are recognized as pathogen-associated molecular patterns (PAMPs) or microbial-associated molecular patterns (MAMPs) by the cells of the innate immunity through the pattern recognition receptors (PRRs). Damage or death-associated molecular patterns (DAMPs) are expressed or released from endangered and damaged host cells and include high mobility group box 1 (HMGB1) and heat shock proteins (HSPs). DAMPs are also recognized by PRRs, resulting in stimulation of the innate immune system following unplanned cell death. These molecules trigger the inflammatory response by activating the mitogen-activated protein kinases (MAPK) and nuclear factor of kappa light polypeptide gene enhancer in B-cells (NF-κB) pathways (Tang et al., 2012) (Figure 1).

Different types of PRRs recognize a wide variety of antigenic threats in the body; they may be localized on cellular or endosomal membranes, or in the cytosol, and serve for signaling. On the other hand, secreted forms may also be present in the bloodstream and interstitial fluids. The PRRs are divided into four families: (i) toll-like receptors (TLR), (ii) nucleotide-binding oligomerization domain-like receptors (NLR), (iii) C-type lectin receptors (CLR), (iv) RIG-1 like receptors (RLR).

TLR and RIG-1 play a major role in recognizing viruses. TLRs are expressed mainly in immune cells and to a lesser extent, in various other cells. Currently, 11 members of the TLR family are known. Each of these is specialized to recognize different PAMPs/MAMPs. For example, TLR3 and TLR9 recognize viruses. TLR3 also controls type I interferon (IFN)-based immunity through interferon regulating factors (IRF). Viral nucleic acids (RNAs) generally serve as PAMPs/MAMPs, and trigger PRRs, including endosomal TLRs 3 and 7 and cytosolic RIG-I-like receptors (RLRs), leading to induction of proinflammatory cytokine-inducing transcription factors such as NF-κB, as well as activation of IRFs that mediate type I IFN-based antiviral response to eliminate the viral agents that threaten the body (Crow et al., 2019; Kumar et al., 2020; Schnappauf et al., 2020). Another pathway for antiviral activity proceeds through RLRs. The RLR family consists of three members: retinoic acid-inducible gene I (RIG-I), melanoma differentiation-associated gene 5 (MDA5), and genetics and physiology lab 2 (LGP2). Additionally, RIG-I and MDA5 have two CARD domains. Upon activation of RLRs by RNA binding, CARD interacts with the adapter mitochondrial antiviral signal protein (MAVS), ultimately leading to the transcription of genes encoding type I IFNs. Type I IFNs further coordinate cellular immune responses against virus infection and are therefore required for antiviral immunity (Zalinger et al., 2015; Barrat et al., 2016; Lucherini et al., 2018; Crow et al., 2019). 

**Figure 2:**
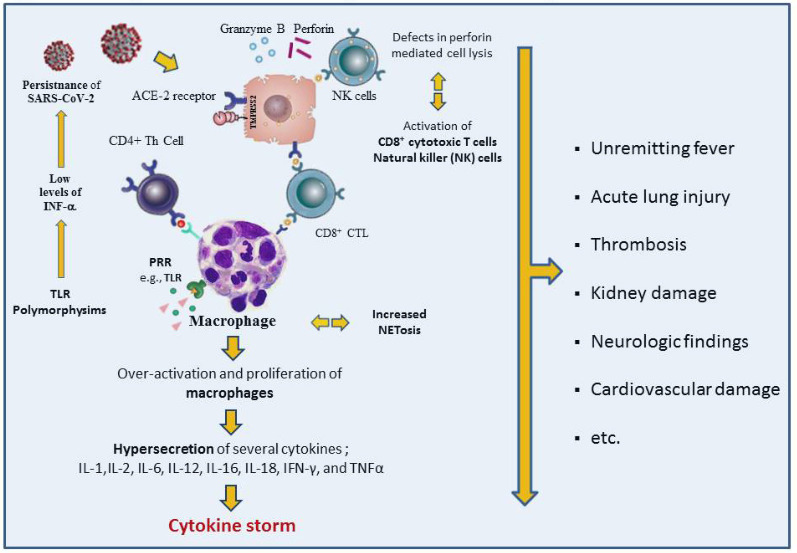
Pathogenesis and clinical findings in COVID-19 induced cytokine storm.

Under normal conditions, virus-infected cells are destroyed by NK cells of innate immunity and CD8+ cytolytic T cells of adaptive immunity using various mechanisms such as perforin-mediated granulysin secretion and direct cytotoxicity. This leads to apoptosis of antigen-presenting cells (APC) and related cytotoxic T cells to prevent unnecessary activation after the antigenic activity ends (Figure 1). If the antigenic stimulation is persistent due to various reasons or there is a genetic or acquired defect in lymphocyte cytolytic activity, NK and cytolytic CD8+ T cells can no longer lyse the infected cells, leading to uncontrolled and continuous activation of innate and adaptive immune cells. As expected, this results in uncontrolled secretion of many proinflammatory cytokines, including TNF-a, IL-1, IL-6, IL-18, and IL-33, causing a cytokine storm. In brief, defects in lymphocyte cytolytic activity continue with increased macrophage activity, uncontrolled immune system activation, and a hyperinflammatory condition called COVID-CS, which causes ARDS and multiorgan failure, and sometimes death (Figure 2) (Huang et al., 2020; Zhang et al., 2020a). However, severe COVID-CS which is one of the major causes of death in COVID-19 occurs only in a minority of the patients. In other words, hyperinflammation develops only in some patients during COVID-19. Probably, virus-related factors and genetically determined or acquired defects in the host immune system, as outlined in the following paragraphs may explain why COVID-CS develops in some patients, although the exact answer is not known (Huang et al., 2020; Samudrala et al., 2020; Soy et al., 2020; Zhang et al., 2020a; Caricchio et al., 2021; Nigrovic et al., 2021).

### 3.1. Virus related factors

Why the antigenic threat created by the virus cannot be eliminated? SARS-CoV and MERS-CoV develop various strategies to stay in the cell longer. SARS-CoV and MERS-CoV can produce double-membrane vesicles that do not have PRRs and can be hidden from host immunity by replicating in these vesicles (Snijder et al., 2006). This may cause antigenic persistence and insufficient viral clearance. The observations that virus inclusion bodies can still be demonstrated in pulmonary alveolar cells and macrophages up to two weeks, despite negative PCR, supports the presence of a problem in viral clearance (Min et al., 2016).

When compared to SARS-CoV, the receptor-binding site (RBS) of the S1 domain in SARS-CoV-2 is highly variable and five amino acids of the six amino acid-region found in this S1 domain differ in the SARS-CoV-2 virus. This provides SARS-CoV-2 with high affinity and optimal binding to ACE-2 receptors (Samudrala et al., 2020). Other than mutations in the RBD of the S protein, the presence of O-linked glycans and polybasic furin cleavage also contributes to the high affinity, rapid transmission, replication, and infectivity of this virus (Andersen KG et al., 2020; Walls et al., 2020).

The persistence of the SARS-CoV-2 virus in the vascular bed may play a role in the pathogenesis of chilblains occurring after COVID-19, and probably in the pathogenesis of MIS-C (multisystem inflammatory syndrome), which may develop after infection in children and resembling Kawasaki syndrome (Colmenero et al., 2020; Schwartz et al., 2021). In addition, the postinfectious complaints and clinical problems, which continue after COVID-19 and are called long COVID, and other similar problems that persist after the acute infection, suggest that there may be a virus or its residue in the body that cannot be cleared (Perego et al., 2020; Ramos-Casals et al., 2021; Writing Committee for the COMEBACK et al., 2021).

Another problem is rapid viral mutations. Although numerous mutations have been reported to date, only five of them are accepted as variants currently. These variants seem to spread more easily and faster than other known variants, which could lead to more COVID-19 cases, more hospitalizations, and potentially more deaths. Studies so far show that despite lower neutralizing antibody titers produced by vaccination permitted in the USA, antibodies may recognize these variants. In other words, although these vaccines provide approximately 10-fold lower neutralizing antibody titers against variants, it is thought that they may be effective (Luchsinger et al., 2021).

### 3.2. Genetic defects in host defences

Genetic polymorphisms of the receptors of antiviral defense may contribute to the severe course of viral infections, including COVID-19. TLR3 polymorphisms have been associated with infectious, autoimmune, and neoplastic diseases (Skevaki et al., 2015). In previous studies, TLR3 polymorphisms are associated with severe hepatitis B and C virus infections; currently, these polymorphisms have also been related to COVID-19 severity (Zhang et al., 2020a; Grolmusz et al., 2021).

Serum IFN-a levels may be found to be very low in some COVID-19 cases due to TLR3 or IRF3 polymorphisms. In addition, anti-IFN-a antibodies may cause very low levels of IFN-a in some patients. Low levels of IFN-a are related to severe COVID-19 pneumonitis. However, innate defects of IRF7-dependent type I IFN immunity were found in only 3.5% of patients. Possibly, other TLRs and related gene polymorphisms also play a role in determining the severity of SARS-COV-2 infection (Zhang et al., 2020a). Interestingly, TLR4, which plays a role in antibacterial defense, can also trigger IFN-a activation and neutrophil extracellular traps (NETS) and contribute to antiviral defense (Khanmohammadi et al., 2021). In addition, testosterone may increase TLR4 production, leading to a higher cytokine response, which may be another mechanism of higher cytokine response in males (Cicco et al., 2020).

An increased number of circulating neutrophils have been identified as an indicator of the severity of COVID-19 (Guan et al., 2020). Neutrophils generate a variety of mechanisms to eliminate invading pathogens, including viruses. They can kill microbes not only through phagocytosis, formation of reactive oxygen species, degranulation, and secretion of antimicrobials, but also through the formation of NETs, and this process is called NETosis. NETs are networks of extracellular neutrophil DNA fibers that bind and kill extracellular pathogens and limit the damage of host cells (Kaplan et al., 2012). NETs are extracellular reticulate fibers composed of DNA and histone and containing enzymes derived from neutrophilic granules such as myeloperoxidase (MPO) and elastase. The NET formation is a type of programmed cell death, different from apoptosis and necrosis. Several antigenic stimuli including viral RNA and proinflammatory cytokines can induce both NETs and NETosis formation. Besides their antimicrobial effects, NETs also play a role in the pathogenesis of a variety of diseases, including SLE, rheumatoid arthritis, diabetes mellitus, and sepsis (Lee et al., 2017). Furthermore, some drugs that can prevent the development of NETosis have been shown to have positive effects on patient survival in the course of sepsis. Recently, Veras et al. showed, there was a high NET formation in tracheal aspirates, lung infiltrates, and peripheral neutrophils of patients with COVID-19, but not in the lungs of patients who died because of heart failure. In addition, they showed that the blocker of the PAD-4 enzyme, which has a critical role in the formation of NETosis, may inhibit the formation of NETs (Veras et al., 2020). The same group also demonstrated that serine protease inhibitors blocking the ACE-2/TMPRSS2 pathway, which has a critical role for SARS-CoV-2 entry to cells, prevent SARS-CoV-2 induced NETs released by neutrophils. These data suggest that NET formation plays a role in the pathogenesis of SARS-CoV-2 and that therapies directed at this pathway may work in preventing lung damage (Cicco et al., 2020).

Decreased anti-SARS-CoV-2 antibody titer, decreased specificity, and decreased neutralizing activity have been reported in patients with MIS-C, a post-COVID-19 life-threatening complication in children, compared with patients with acute COVID-19 respiratory infection. The authors claimed that younger people had dense naive T cells located in various parts of the body and prevented infection in organs such as the lungs with a strong immune response and that the weaker but ongoing infection in other regions could lead to MIS-C by getting stronger over time (Jia et al., 2020; Weisberg et al., 2020). The presence of the SARS-CoV-2 virus detected in the vascular bed in children may also explain the delayed complications (Colmenero et al., 2020; Schwartz et al., 2021). SARS-CoV-2 virus, which takes advantage of the abovementioned mechanisms and various disorders in the immune system, causes infection in the upper and lower respiratory tract where the expression of ACE-2 receptor is intense, followed by uncontrolled infection and proinflammatory cytokine release, resulting in hyper inflammation, serious organ damage, ARDS, DIC, and thrombotic complications (Figure 2).

COVID-19 is also associated with a significant risk of micro- and macrovascular thrombotic complications such as venous thromboembolic disease, pulmonary embolism, and stroke, which are also associated with multiorgan failure and increased mortality. In addition to classical prothrombotic factors, immune mechanisms also play a role in the formation of thrombosis. TNF-ɑ, one of the increased cytokines in the course of COVID-19, triggers an increase in plasminogen activator inhibitor-1, which inhibits tissue plasminogen activator, which leads to a decrease in plasmin activity, reducing fibrinolysis. Complement activation, cell lysis, von Willebrand factor formation, formation of NETosis, increasing tissue factor synthesis, and prothrombin activity may also contribute to thrombotic tendency (Loo et al., 2021). In brief, inadequate eradication of the SARS-CoV-2 virus due to various combinations of viral factors and genetic defects in host immunity may cause the occurrence of COVID-CS in some patients. 

## 4. Diagnosis of cytokine storm in COVID-19

Diagnosis of COVID-19 can be made by clinical manifestations, radiologic evidence of viral pneumonia, and PCR testing for COVID-19. On the other hand, early diagnosis of COVID-CS is of particular importance, because patients can be critically ill and delays in diagnosis can lead to poor outcomes. Unfortunately, there is no single pathognomonic clinical finding or laboratory test to diagnose this life-threatening condition (Lei et al., 2020). The criteria used for HLH and MAS are not suitable for COVID-CS (Soy et al., 2021). Recently Caricchio et al. proposed a preliminary set of criteria for predicting COVID-CS (Caricchio et al., 2020). They defined five obligatory criteria and a three more cluster sets containing various parameters. According to those preliminary set of criteria, patients should have at least one of the items contained in all three cluster sets, in addition to these obligatory criteria (Table). Although those preliminary set of criteria may be useful in some patients whose general condition is deteriorating, we believe that it is too early to use them as definite diagnostic criteria; further studies are needed. Simply put, COVID-CS should be considered in a patient with a confirmed diagnosis of COVID-19, if there is a rapid deterioration of the general condition, persistent fever lasting than three days, progressive increase in serum CRP, ferritin, and D-dimer values. This diagnosis and management should be guided by a committee consisting of at least one of the following: rheumatologist trained in internal medicine, hematologist, clinical immunologist, pneumologist, a specialist in infectious diseases, and specialist of intensive care.

**Table:** Predictive criteria for cytokine storm in COVID-19 proposed by Caricchio et al. (2021)*.

Entry criteria (must be all met)	Cut-off values	Comments
• +Signs/symptoms of COVID-19 • ±RT-PCR positive for COVID-19 • +GGO by HRCT (or chest X-ray) • Ferritin • C-reactive protein	>250 ng/mL>4.6mg/dL	RT-PCR positivity was not mandatory due to the high percentage of false negatives (15%). Ferritin and CRP appeared as strong indicators of acute inflammation in COVID-CS**.
AND (one variable from each cluster)		
Cluster I Albumin Lymphocyte (%)Neutrophil(absolute)	<2.8 g/dL10.211.4 K/mm3	Neutrophils and monocytes were found to be significantly increased in COVID-CS cases, suggesting an active role of innate immunity in COVID-CS. On the other hand, lymphocytes declined nearly to half of the lower normal limit, suggesting that adaptive immunity in COVID-CS was functionally depleted. Some authors suggested additionally parameters such as NLR, MLR***.
Cluster II ALT AST D-dimers LDH Troponin I	>60 U/L>87 U/L>4,930 ng/mL>416 U/L>1.09 ng/mL	ALT and AST indicative of liver cell damage were elevated to nearly twice higher normal limit; Six times higher levels of D-dimers implicated endothelial damage and thrombosis formation. Increased LDH was a sign of cell death, while moderately elevated troponin levels suggested damage of cardiovascular system.
Cluster III Anion gap ChloridePotassium BUN:creatinine ratio	<6.8>106 mmol/L >4.9 mmol/L >29 ratio	These results suggested a prerenal imbalance and kidney damage. Taken together, they reflected systemic tissue damage affecting many organs in COVID-CS.

## 5. Management of cytokine storm in COVID-19 infection

Management of COVID-19 includes antiviral agents to inhibit SARS-CoV-2 replication as well as treatment of the potential complications including cytokine storm using appropriate immunosuppressive and immunomodulatory drugs to reduce systemic inflammation. As mentioned above, COVID-CS is a hyperinflammatory state similar to cytokine storms developing in HLH or CAR-T induced cytokine release syndrome. However, there are also differences, including treatment approaches. The earlier the diagnosis of CS, the more effective the treatment is. The rationale of the treatment of COVID-CS, which represents a hyperinflammatory state, is to suppress the inappropriate immune response. IL-6, IL-1, IFN-g, TNF-a, IL-8, and IL-18 play a critical role in the development of the cytokine storm but there may be additional contributory cytokines.

Corticosteroids (CCS) and targeted therapies for certain cytokines, especially IL-6R antagonists including tocilizumab (TCZ) and IL-1 antagonists including anakinra are most commonly used for the treatment of cytokine storm. On the other hand, TNF inhibitors such as infliximab and adalimumab, IFN-γ inhibitors such as emapalumab, and Janus kinase inhibitors such as baricitinib and ruxolitinib are among other agents investigated for this purpose. However, the use of antiinflammatory therapy in severe COVID-CS poses a critical issue. Whatever the antiinflammatory treatment is, the timing is of utmost importance. Otherwise, trying to reach clinical improvement through suppression of excessive immune responses, might paradoxically decrease the clearance of the virus from the body and increase the risk of secondary bacterial infections (Kim et al., 2021). Finally, it should be noted that hyperinflammation in cytokine storm is accompanied by a hypercoagulable state. Therefore, performing thrombosis prophylaxis using an appropriate agent such as enoxaparin is also necessary (Kim et al., 2021). 

Corticosteroids: With appropriate dosing and timing of administration given, CCS treatment is extremely important in the management of the cytokine storm. Early administration of CCSs may inhibit the initiation of the body’s immune defense mechanism, thereby increasing the viral load and ultimately leading to adverse consequences. Indications for CCS treatment include the presence of predictive criteria for cytokine storm as outlined above. Severe and critical clinical state, persistent fever (>39 °C), acute hypoxemic respiratory failure, rapid deterioration suggested by CT-scan findings (more than 50% of the infected area on CT-scan images within 48 h), the requirement for mechanical ventilation, and presence of relevant laboratory markers require CCS treatment. Inhibition of excessive inflammation through timely administration of CCSs in the early stage of inflammatory cytokine storm effectively prevents the occurrence of ARDS and protects the functions of the patients’ organs (Shang et al., 2020). RECOVERY trial is a cornerstone controlled, open-label study investigating the effectivity of oral or intravenous dexamethasone 6 mg daily for up to 10 days, compared to usual care alone. The primary outcome was 28-day mortality. A total of 2104 patients hospitalized with COVID-19 were assigned to receive dexamethasone and 4321 to receive usual care. The use of dexamethasone resulted in significantly lower 28-day mortality than usual care among those who were receiving either invasive mechanical ventilation or oxygen alone at randomization but not among those receiving no respiratory support. However, there was no evidence that dexamethasone provided any benefit among patients who were not receiving respiratory support at randomization, and the results were consistent with possible harm in this subgroup. This observation might be explained that those patients probably represented the subgroup without cytokine storm, in whom dexamethasone treatment deteriorated viral clearance (The RECOVERY Collaborative Group et al., 2021).

The median duration of dexamethasone treatment in that study was only seven days. An early course of three to five days of CCSs is generally appropriate. However, many experts use higher doses of CCSs (methylprednisolone 1–2 mg/kg/day or equivalent doses of dexamethasone) for longer periods in patients with severe ARDS. If higher doses of CCSs are used, the dose may be reduced to 6 mg dexamethasone daily or equivalent as soon as improvement occurs. Dexamethasone has a long biological half-life with its auto-taper property and thereby prevents rebound inflammation. The beneficial effect of dexamethasone was also reported in CoDEX randomized clinical trial. Among patients with COVID-19 and moderate or severe ARDS, use of intravenous dexamethasone plus standard care compared with standard care alone resulted in a statistically significant increase in the number of ventilator-free days over 28 days (Tomazini et al., 2020).

The final point statement confirming the effectivity of CCS treatment was the result of the prospective metaanalysis of clinical trials of critically ill patients with COVID-19, performed by Sterne et al. (2020). They reported that administration of systemic CCSs was associated with lower 28-day all-cause mortality, compared with usual care or placebo (WHO Rapid Evidence Appraisal for COVID-19 Therapies (REACT) Working Group et al., 2020). 

On the other hand, we believe that if anticytokine treatment targeting IL-6 or IL-1 will be used later, very high doses of CCS should be avoided to reduce potential complications of secondary opportunistic bacterial or fungal infections. 

Inhibition of interleukin-6 signaling: IL-6 is an important proinflammatory cytokine secreted from macrophages and has pleiotropic effects. It is induced by infection or tissue injury and promotes the production of various acute-phase proteins in hepatocytes and induces the differentiation of immune cells, such as B and T cells. Besides, IL-6 is involved in the metabolism of iron by inducing hepcidin which decreases iron absorption, to make a microenvironment prohibitive against infection. Taken together, in priming inflammatory responses and activating adaptive immunity against infection or injury IL-6 is a key cytokine (Tanaka et al., 2014). Not only significantly elevated serum IL-6 levels were noted in patients with COVID-19, but predictivity of high IL-6 for poor outcomes in COVID-19 was also reported. It was reported that nonsurvivors had higher IL-6 levels than survivors, and high levels of IL-6 predicted the risk of requiring mechanical ventilation (Zhou et al., 2020). Furthermore, a recent prospective cohort study indicated that high levels of IL-6 and D-dimer reflected systemic inflammation and thrombotic condition and predicted in-hospital mortality of COVID-19 (Cummings et al., 2020).

TCZ is a humanized anti-IL-6 receptor monoclonal antibody, inhibiting IL-6. TCZ is currently used not only for the therapy of various autoimmune rheumatological diseases including rheumatoid arthritis, temporal arteritis, and Takayasu arteritis but also for the treatment of the cytokine storm which may be induced by CAR-T treatment. Based on these observations and the abovementioned role of IL-6 in patients with severe SARS-CoV-2 infection complicated with cytokine storm and ARDS, TCZ treatment was also tried in these patients. 

Retrospective studies from China reported the resolution of fever and hypoxemia, and improvement in serum CRP levels and pulmonary CT findings (Xu et al., 2020). Other studies and clinical trials also supported these observations (Toniati et al., 2020; Fu et al., 2020; Zhang et al., 2020b; Colaneri et al., 2020). A recent retrospective cohort study of 1351 patients with COVID-19 and pneumonia showed that TCZ significantly reduced the mechanical ventilation risk or death (Campochiaro et al., 2020). In an observational study of 154 patients requiring mechanical ventilation, TCZ reduced the risk of death by 45% (Somers et al., 2020). 

In the EMPACTA trial, 389 hospitalized patients with COVID-19 pneumonia who were not receiving mechanical ventilation, were randomized and TCZ was reported to reduce the likelihood of progression to the composite outcome of mechanical ventilation or death (Salama et al., 2021). 

However, there are also studies with negative results. In a randomized, double-blind, placebo-controlled trial, Stone et al. reported that TCZ was not effective for preventing intubation or death in moderately ill hospitalized patients with COVID-19 (Stone et al., 2020).

For the treatment of cytokine storm, the recommended TCZ dose is 8 mg/kg IV as single or divided two doses by 12–24 h intervals (maximum dose 800 mg). Similar to CCSs, admission of TCZ should also be on time; serum procalcitonin levels should be normal and the presence of any active infection should be excluded. Because the most fearful adverse effect of TCZ is the tendency for general infections, as also reported in clinical studies. Since serum CRP levels and some of the clinical signs of infections are depressed as the result of IL-6 inhibition, diagnosis of infections may be difficult. Other potential adverse effects of TCZ include hepatotoxicity, hyperlipidemia, leucopenia, and the possibility of diverticulitis.

Inhibition of interleukin-1 signaling: IL-1 family is a group of 11 cytokines that plays a central role in the regulation of immune and inflammatory responses to infections or various non-infectious insults. IL-1α, IL-1β, and IL-18 are outstanding members of this family. Following inflammasome activation, the caspase 1 enzyme converts pro-IL-1β and pro-IL-18 to IL-1β and IL-18, respectively (Dinarello et al., 2013) (Figure 2). SARS-CoV-2 may cause not only a cytokine storm where IL-1β and IL-18 play important roles, but also may cause pyroptosis, a form of cell death that is triggered by proinflammatory signals and associated with inflammation. A cardinal feature of pyroptosis is the requirement for caspase-1 activation, and cells undergoing pyroptosis release increased amounts of IL-1β and IL-18 (Ferreira et al., 2021). 

Taken together, inhibition of IL-1β may be helpful in patients developing a cytokine storm (Mehta et al., 2020). IL-1 Receptor agonist (IL-1Ra) is also a member of the IL-1 family and serves as the natural inhibitor of both IL-1α and IL-1β. Anakinra is the recombinant form of IL-1Ra (rHIL-1Ra) and is the first IL-1 blocking biologic agent produced. Anakinra blocks the binding of both IL-1α and IL-1β to IL-1R, thereby inhibiting the proinflammatory effects of both IL-1α and IL-1β. Anakinra is currently used in the treatment of FMF and some other autoinflammatory diseases. Since anakinra is a modified form of the physiologic inhibitor, it may be expected to be rather safe relative to other agents in the same line. However, the tendency to infections is also a concern for anakinra. Another IL-1 inhibitor, canakinumab which is a human monoclonal antibody neutralizing IL-1ß, has also been used for similar indications with the advantage of prolonged action (Dinarello et al., 2013).

A few studies have suggested beneficial effects with the use of anakinra in COVID-19 (Huet et al., 2020; Cavalli et al., 2021). A recent retrospective cohort study of patients with COVID-19 and ARDS showed that high-dose anakinra could be used safely and improved respiratory function. Another prospective cohort study of patients with severe COVID-19 pneumonia demonstrated that anakinra reduced the need for mechanical ventilation and mortality without serious side effects (Huet et al., 2020). Although both studies showed promising results for anakinra on severe COVID-19, there are also negative clinical trials. In the multicentre, open-label, Bayesian randomized CORIMUNO-ANA-1 clinical trial, anakinra did not improve the outcomes, which were defined as the proportion of patients who died or needed non-invasive or mechanical ventilation by day 4 and survival without the need for mechanical or noninvasive ventilation (including high-flow oxygen) at day 14 (The CORIMUNO-19 Collaborative Group et al., 2021). Further randomized clinical trials are obviously needed. 

Anakinra has a short half-life (about 4–6 h) and the recommended dose used in FMF (100–200 mg/day SC) is not sufficient for the treatment of the cytokine storm occurring in COVID-19. Although even in some clinical randomized COVID trials, anakinra was given for 3–5 days at lower doses, recent data favors the use of higher doses of anakinra (10 mg/kg per day divided twice daily) SC or with IV infusion until clinic benefit was achieved (King et al., 2020). Commonly, a daily titration of the anakinra based on the clinical and laboratory parameters is recommended as expert opinion. 

The bioavailability of SC injections is 95% with a half-life of 4–6 h. In the presence of renal failure (GFR < 30 mL/min), the dose should be reduced. However, the hepatic disease does not require dose adjustment. Unlike TCZ, anakinra does not inhibit CRP synthesis directly, therefore serum CRP levels can be used to follow up systemic acute phase response (King et al., 2020). 

Comparison of IL-6 versus IL-1 inhibition in cytokine storm of COVID 19: In a cohort study performed by Cavalli et al. (2021), IL-1 and IL-6 inhibition were compared with standard management in patients with COVID-19 developing respiratory insufficiency and hyperinflammation. The primary endpoint was survival, and the secondary endpoint was a composite of death or mechanical ventilation. Among 392 patients included in this study, 275 received only standard care, while 62 received anakinra and 55 received tocilizumab or sarilumab. The main finding of this study was that IL-1 inhibition with anakinra, but not IL-6 inhibition with, significantly reduced mortality in the overall population of in-hospital patients with COVID-19, respiratory insufficiency, and hyperinflammation. Protective effects of IL-6 inhibition were only observed only in patients with very high serum CRP at baseline, whereas both IL-1 and IL-6 inhibitors were more effective in patients with low serum lactate dehydrogenase at baseline. There was no difference in adverse clinical outcome risk both in patients treated with IL-1 inhibition or IL-6 inhibition, compared to the rest of the patients who did not receive interleukin inhibitors (Cavalli et al., 2021).

Based upon these findings, the authors suggested that the potential benefit of interleukin inhibition is highest in the early phases of the disease, characterized by rampant inflammatory activation but progressively fades in more advanced stages characterized by extensive disease burden and tissue damage. The hypothesis of the greater efficacy of anakinra in early phases of disease is in line with previous studies which reported increased survival in macrophage activation syndrome following early intervention with IL-1 blockade (Cavalli et al., 2021).

If IL-1 blockade is really more beneficial than IL-6 inhibition, this may be due to the upstream expression of IL-1 compared to IL-6. In other words, blocking IL-1 signaling may also indirectly block IL-6. Another possibility is related to the endotheliopathy associated with COVID-19 and the release of IL-1α, which may be inhibited by anakinra (Cavalli et al., 2021). The question of whether immunomodulatory therapies improved survival in patients with COVID-19 cytokine storm was also investigated by Narain et al., on behalf of the Northwell COVID-19 Research Consortium. The retrospective comparative survival analysis showed that the combination of CCSs with TCZ had superior survival outcomes when compared with standard treatment as well as treatment with CCSs alone or in combination with anakinra. Furthermore, CCS use either alone or in combination with TCZ or anakinra was associated with reduced hospital mortality CCS compared with patients receiving standard treatment (Narain et al., 2021).

Langer-Gould et al. (2020) also examined outcomes among patients who were treated with anakinra or TCZ for COVID-19 -related cytokine storm. In this retrospective cohort study, initial data showed the superiority of anakinra. However, after accounting for differences in disease severity at treatment initiation, this apparent superiority of anakinra over TCZ was no longer statistically significant. They concluded that prompt identification and treatment of COVID19 cytokine storm before intubation might be more important than the specific type of antiinflammatory treatment. They also noted that randomized controlled trials of targeted anticytokine treatments and CCSs should report the duration of cytokine storm in addition to clinical severity at randomization (Langer-Gould et al., 2020).

Since plasma concentrations of cytokines including IL-1 and IL-6 exhibit diurnal variations, reaching higher concentrations in the afternoon than in the morning, the timing of drug administration in cytokine-targeted therapy is also important (Kim et al., 2021).

Inhibition of TNF-α signaling: TNF-α is an important proinflammatory cytokine primarily produced by activated macrophages and contributes to the COVID-19 hyperinflammatory state. Besides, TNF-α induces NETs release, or NETosis, whereby neutrophils release sticky extracellular traps that help limit the spread of infection as mentioned above. SARs-CoV-2 virus directly promotes NETosis, thereby activating both platelets and the clotting cascade, leading to thrombi. Besides, NETosis induces pulmonary epithelial cell death, leading to alveolar damage and fibrosis in COVID-19 patients. Of relevance, anti-TNF therapy may not only inhibit the hyperinflammatory state but also may reduce NET formation in COVID-19 patients (Robinson et al., 2020). A recent interesting study showed that TNF-α mediated acute lung injury was reduced by using an aptamer targeting TNF-α (Lai et al., 2019). Since acute lung injury is also a characteristic finding in COVID-19, this study also supports the possible benefit of anti-TNF agents in the treatment of the cytokine storm of COVID-19. However, current data is limited, and there are a few small ongoing clinical trials, including CATALYST, ACTIV-1, and AVID-CC, that infliximab and adalimumab are investigated for this purpose (Robinson et al., 2020). On the other hand, observational human data, including small case series and larger databases, have shown a possible benefit of maintenance anti-TNF therapy in patients with COVID-19 (Brenner et al., 2020; Gianfrancesco. et al., 2020; Mahil et al., 2021). After controlling for important confounders, patients using anti-TNF therapy for the underlying rheumatic disease were found to have significantly less hospitalization need related to COVID-19, compared to those without anti-TNF inhibitor therapy. Safety of maintenance anti-TNF treatment was also reported in cohorts of patients with inflammatory bowel disease (IBD). However, selection bias and channeling bias (confounding by indication) should be considered while interpreting this data. Possibly, patients with certain characteristics that put them at higher risk for infection are not prescribed anti-TNF agents (Robinson, et al., 2020). In brief, although observational data on anti-TNF use that must be interpreted with caution are suggestive of a therapeutic benefit in patients with COVID-19, results of randomized controlled trials (RCTs) are needed to confirm this benefit. 

Inhibition of IFN-γ signaling: IFN-γ is secreted mainly by macrophages, NK cells, and T cells, and is among the major effector cytokines in COVID-CS. Although the use of emapalumab, an anti- IFN-γ monoclonal antibody to treat primary HLH was approved by the FDA in 2018, and several clinical trials showed clinical efficacy without severe side effects, no significant trials support the use of IFN-γ inhibitors in COVID-19 yet (Kim et al., 2021).

Inhibition of JAK/STAT pathway: The Janus kinase-signal transducer and activator of transcription (JAK/STAT) pathway is important in mediating the effects of various cytokines and growth hormones. JAK inhibition might effectively suppress the cytokine storm because it can nonselectively inhibit the activity of many cytokines (Seif et al., 2020). However, inhibition of type I IFNs may also impede antiviral immunity (Favalli et al., 2020). On the other hand, JAK inhibition in COVID-19 may also reduce the entry of SARS-CoV-2, in the early phase of infection. SARS-CoV-2 enters the body through ACE-2 on alveolar type 2 cells in the lungs, and several regulators including AP2-associated protein kinase-1 (AAK1) are involved in mediating endocytosis and intracellular transport through ACE-2. Several JAK inhibitors including baricitinib, ruxolitinib, and fedratinib, are inhibitors of AAK1. Therefore, they may impede the cellular entry and proliferation of SARS-CoV-2. Since tofacitinib cannot inhibit AAK1, this agent is not recommended for this purpose (Richardson et al., 2020; Seif et al., 2020; Kim et al., 2021). Based upon this basic data, Stebbing et al. (2020) suggested that baricitinib 2–4 mg daily may be combined with antiviral treatment in severe COVID-19 infections. A recent multicentered RCT of ruxolitinib in patients with COVID-19 noted faster clinical improvement with the drug, although the results lacked statistical significance (Cao et al., 2020a). Another multicentered retrospective study demonstrated that baricitinib reduced the rate of intensive care unit admission and fatality and increased discharge rates (Cantini et al., 2020). Besides the tendency for general infections, inhibition of antiviral type I IFN production remains a disadvantage as pointed out above. 

Type I interferon-based therapies: Physiologic antiviral defense performed by type I interferons including IFN-a and IFN-b seems to be inhibited in COVID-19 infections. The reduction in type I IFNs, may be a consequence of generalized immunosuppression induced by high viral load. It has been shown that severe and critical patients displayed lower activity and the diminished response of type I IFNs compared to mild to moderate patients (Bastard et al., 2020; Zhang et al., 2020a). Since coronavirus inhibits the production of IFN-β1-a from inflammatory cells, usage of IFN-β1-a may be an effective option to increase antiviral immunity (Kow et al., 2020). Baghaei et al. (2021) conducted a retrospective study in adult patients hospitalized with the diagnosis of COVID-19. They compared the combination of three doses of 12 million international units of IFN-β1-a plus Lopinavir 400 mg and Ritonavir 100 mg every 12 h in 152 patients with only Lopinavir/Ritonavir combination in 302 age- and sex-matched control patients for 14 days. All patients received common standard care. The all-cause mortality rate in the case and control groups was 11% and 13%, respectively, with no significant difference between the two groups. These findings were consistent with the interim findings from the World Health Organization’s Solidarity Trial investigating the efficacy of four repurposed antiviral drugs including IFN-β1-a (WHO Solidarity Trial Consortium et al., 2020). The Solidarity Trial also reported no significant difference in the risk of death between patients with COVID-19 who received IFN-β1-a compared to the control group. Although these results are disappointing for IFN-β1-a, Kow et al. commented that it might be premature to discard interferon-based therapies in our armamentarium against COVID-19 (Kow et al., 2020). They reported that interferon-based therapies might only be effective for selected patients having deficiencies in type I interferons, who harbored mutations in the genes related to the toll-like receptor 3 and interferon-I signaling pathways, or those who had autoantibodies that recognized type I interferons. The route of administration of interferon-based therapies may also play a factor. Since the bioavailability via the subcutaneous route is only approximately one-third of that via the intravenous route, the intravenous route of administration can optimize the distribution to the pulmonary vasculature. Indeed, nebulized interferon-based therapy may even provide more targeted actions, and thus more favorable clinical responses. A randomized controlled trial reported that patients with COVID-19 who received inhaled nebulized IFN-β1-a had greater clinical improvement compared to control groups (Monk et al., 2020). In addition, the timing of treatment is critical where interferon-based therapy should preferably be administered in the early stage of the disease. We also agree with Kow et al. and believe that future trials involving interferon-based therapies would give better results if selected patients with concurrent deficiencies in type I interferons were selected, and intravenous or inhalational route of administration is chosen in the early course of the disease.

Therapeutic plasma exchange (TPE): Besides inhibiting cytokines directly, cytokine removal by TPE, blood purification using continuous renal replacement therapy or extracorporeal cytokine removal are other options. TPE may also be considered for the treatment of COVID-CS. Most of the data about TPE comes from studies from secondary HLH and severe sepsis and is somewhat controversial. However, rapid, and nonselective removal of cytokines may help reduce the unfavorable effects of the cytokine storm and may improve the function of monocytes and/or macrophages, thereby reversing immune-mediated damage to the lungs. However, there are several clinical issues with the use of TPE in COVID-CS, including risks of bleeding, catheter infection, electrolyte imbalances, and unpredicted anaphylactic shock due to the use of blood materials. Besides, the use of non-convalescent plasma as a substitution fluid could reduce protective antibodies against SARS-CoV-2. Finally, the cost and technical problems and need for qualified staff are important issues. Therefore, on-time TPE may be considered only in selected cases presenting with COVID-CS, and convalescent plasma should be preferred as the substitution fluid (Kim et al., 2021).

Convalescent plasma: The rationale for using convalescent plasma is to provide passive immunization to control the evolution of the disease until a specific immune response is established in the infected person. In general, early use of convalescent plasma, before critical illness develops, may be an important predictor of the efficacy of passive immunotherapy for that pathogen (Katz et al., 2021). Observational studies have consistently shown that convalescent plasma has an adequate safety profile in patients with COVID-19. An exploratory analysis in 4330 patients showed no significant difference in 7-day mortality between patients who received high-titer convalescent plasma and those who received low-titer convalescent plasma in the overall population. However, the superiority of high-titer convalescent plasma was seen in the predefined subgroup of nonintubated patients. In this subgroup, 20% lower 7-day mortality was 14%, versus 11%, in patients receiving high titer versus low titer convalescent plasma, respectively (p = 0.03) (Huang et al., 2020). A similar efficacy analysis from the Mayo Clinic included 3082 participants and reported that the 30-day mortality rate was 29.1% in the low-titer group and 24.7% in the high-titer group; the difference did not reach statistical significance (Joyner et al., 2020). However, a post hoc subgroup analysis in this study also suggested a benefit of high-titer plasma in patients who received plasma within 3 days after COVID-19 diagnosis, probably confirming the importance of early therapy. 

Recently Simonovich et al. (2021) randomly assigned hospitalized adult patients with severe COVID-19 pneumonia in a 2:1 ratio to receive convalescent plasma or placebo. The primary outcome was the patient’s clinical status 30 days after the intervention, as measured on a six-point ordinal scale ranging from total recovery to death. A total of 228 patients were assigned to receive convalescent plasma and 105 to receive placebo. At the end of 30 days, no significant differences were observed in clinical status or overall mortality between patients treated with convalescent plasma and those who received placebo (Simonovich et al., 2021).

While using convalescent plasma, possible adverse effects including fluid overload, transfusion-associated acute lung injury, and allergy should be considered. However, the benefits of convalescent plasma would outweigh its risks; and observational studies have been more positive than randomized trials. This is possibly due to the use of convalescent plasma to critically ill patients in the intensive care unit in clinical trials. However, convalescent plasma should be used as early as possible in the course of infection (preferably within 3 days after diagnosis) to achieve the best outcomes.

This statement was also confirmed by Libster et al. (2021), who investigated whether early high-titer plasma therapy might prevent severe Covid-19 in older adults. They made a double-blind trial, comparing 250 mL of convalescent plasma with an IgG titer greater than 1:1000 against SARS-CoV-2 spike (S) protein with saline placebo in patients who were 65 to 74 years of age and had prespecified coexisting conditions and in patients who were 75 years of age or older with or without coexisting conditions. The patients received convalescent plasma or placebo less than 72 h after symptom onset. They observed no serious adverse events and concluded that early administration of high-titer convalescent plasma against SARS-CoV-2 to mildly ill infected older adults reduced the progression of COVID-19. Younger high-risk patients and certain immunodeficient patients in early disease should be considered convalescent plasma as well (Libster et al., 2021).

Intravenous immunoglobulin (IVIG): IVIG is a blood product prepared from the serum pooled from thousands of healthy donors. IVIG preparations mainly consist of IgG1 and IgG2 subclasses, however, trace amounts of IgA and IgM may also be found. In clinical practice, IVIG is used in patients with immune deficiencies for the treatment of infectious diseases, as well as in treatment-resistant patients with autoimmune diseases as an immunomodulatory agent. However, in COVID-19, the actual role of IVIG is not to boost the immune system. In the presence of coexisting immunodeficiency, IVIG serves to solve this problem, but the most important action of IVIG is pleiotropic immunomodulating actions, involving both innate and adaptive immunity, to suppress a hyperactive immune response that is seen in some patients (Tzilas et al., 2020).

IVIG is generally used 0.3–0.5 gr/kg/day for five days. Besides case reports, retrospective case series, and small observational studies, there are also ongoing randomized, open‐label, controlled, single, or multicenter trials (Cao et al., 2020b; Moradimajd et al., 2021). In a retrospective study of 58 cases with severe or critical illness due to COVID-19, early administration of IVIG was associated with reduced ventilator use, reduced hospital and intensive care unit length of stay, and improved 28-day mortality (Xie et al., 2020). Similarly, Herth et al. reported that IVIG treatment not only improved the clinical state of patients with moderate to severe COVID-19 infection but also reduced the length of hospital stay. They observed that the earlier IVIG was given, the shorter the length of hospital stay was (Herth et al., 2020). Since the initiation of the cytokine storm generally takes place 5–7 days after initiation of symptoms and represents the time window in which immunomodulation is likely to be most beneficial, selecting the appropriate patients and early administration of IVIG are obviously very important (Tzilas et al., 2020). 

Although the general impression of IVIG treatment is favorable, there are many limitations in evaluating clinical improvements using IVIG therapy in patients with COVID‐19. These limitations include a small number of patients, the combined use of IVIG with antimicrobial drugs and corticosteroids, variations in dosage and timing for IVIG injection, and lack of careful evaluation of the clinical results. Moradimajd et al., 2021). Therefore, it is not possible to make a correct judgment on the therapeutic effect of IVIG in patients with COVID‐19. 

Finally, possible adverse effects of IVIG should also be considered. The mild adverse effects include flushing, headache, malaise, fever, chills, fatigue, and lethargy. Some of the serious side effects of IVIG therapy include hemolytic anemia, thrombosis, arrhythmia, aseptic meningitis, renal impairment, and transfusion-related acute lung injury (Moradimajd et al., 2021). Anticoagulation and hydration should not be overlooked since COVID-19 patients have an increased tendency towards thrombosis during IVIG treatment.

Venous thromboembolism (VTE) prophylaxis: Most patients with a cytokine storm due to COVID-19 infection seem to be extremely hypercoagulable. This would support a potential role for VTE prophylaxis in COVID-19 infection. Enoxaparin 30 SC mg bid is suggested as the preferred dose for VTE prophylaxis in critically ill patients with COVID-19. Enoxaparin 30 mg SC bid should also be considered for VTE prophylaxis in hospitalized ward-based patients. Higher doses of anticoagulant prophylaxis (enoxaparin 0.5 mg/kg BW SC bid) may be considered in patients with moderately elevated D-dimer (1500 ng/mL) and for patients with weight above 100 kg or BMI above 40 kg/m2. Factor Xa level 4 h after the third dose may be checked, targeting a level of 0.5–0.8 IU/mL (Bikdeli et al., 2020).

Colchicine: Colchicine is an antiinflammatory and immunomodulatory agent, commonly used for the treatment of gout, FMF, and Behçet’s syndrome for a long time. The main action of colchicine is to impede the function of neutrophils, and it has the effect of inhibiting IL-1ß activity by inhibition of the inflammasome complex. Colchicine was suggested to be useful for the treatment of some complications of COVID-19 infection, based on its ability to inhibit IL-1 production. however phase 3, multicenter, randomized, double-blind, placebo-controlled multicenter COLCORONA (colchicine coronavirus SARS-CoV-2) trial failed to show the efficacy of colchicine in adult patients diagnosed with COVID-19 infection; but, these results were not confirmed and remain elusive (Salah et al 2021). In addition, the colchicine arm of the long-standing RECOVERY trial was suspended for new patients because of ineffectiveness. 

Other therapeutic options: Some clinicians proposed to use stem cells or low-dose radiation therapy of the lungs to control the hyperinflammatory state to establish an immune-modulation through the regulatory effects on specialized immune cells in severe COVID-19. Several drugs including peroxisome proliferator-activated receptors (PPARs) agonists, cyclooxygenase (COX) inhibitors, and sphingosine-1-phosphate receptor 1 (S1P1) which have been experimentally shown to be effective in reducing cytokine storm associated with influenza infection, may also be considered to use for severe COVID-19 in the future. Use of nanomedicine including liposomes or synthetic nanoparticles to modulate macrophage dysfunction and to inhibit dysregulated macrophages can be a novel therapeutic approach for the treatment of the cytokine storm (Kim et al., 2021). However, all of these options are currently experimental, and the data is limited.

Although it may not play a direct role in the treatment of hyperinflammatory state in COVID-19, recent trends for inhibition of coronavirus cell entry deserve attention. It has been demonstrated that ACE-2 is the cellular receptor that SARS-CoV-2 uses for entering the cell. Recently, a TMPRSS2 inhibitor was approved for clinical use for blocking entry and might constitute a treatment option (Hoffmann et al., 2020). 

## 6. Discussion and conclusion

It seems that the COVID-19 pandemic will occupy the world agenda further for a long time. Although it does not cause serious illness in the majority of the patients, especially elderly patients with comorbidities are prone to a more severe and possibly lethal disease course caused by a hyperinflammatory state known as COVID-CS. 

Although exact pathogenesis of COVID-CS is not known, insufficient viral clearance due to viral factors and genetic defects in host defense, resulting in strong cytokine response and hyperinflammation play critical role (Snijder et al., 2006; Min et al., 2016; Colmenero et al., 2020; Schwartz et al., 2021).

The diagnosis of COVID-19 is based on relatively constant clinical signs, findings, laboratory tests, and imaging techniques, while the diagnosis of COVID-CS depends on emerging findings during the clinical course. We believe that COVID-CS should be considered in a patient with a confirmed diagnosis of COVID-19, and a rapid deterioration of the patient with the presence of persistent fever lasting more than three to four days, progressive increase in serum CRP, ferritin, and D-dimer values. 

Therapy of COVID-19 consists of antiviral agents to inhibit SARS-CoV-2 replication as well as treatment of COVID-CS with appropriate immunosuppressive and immunomodulatory drugs to reduce the systemic inflammation as the main cause of organ damage. CCS and targeted therapies for cytokine blocking, such as IL-6R antagonists and IL-1 inhibitors are most commonly used for the treatment of COVID-CS. Independent of the choice of antiinflammatory treatment, the timing to initiate these drugs is very important. Additionally, anticoagulant therapy should also be initiated because of tendency towards hypercoagulability (Loo et al., 2021).

In conclusion, early diagnosis and appropriate effective treatment of COVID-CS are of vital importance, and multidisciplinary approach is obviously necessary.

## Ethics approval 

This is a review article and no ethical approval was required.

## Contribution of authors

This article was designed and written by Mehmet SOY, G**ö**khan DEM**İ**R, and Pamir ATAG**Ü**ND**Ü**Z. It was reviewed by all authors to structure the final version for publication. The authors accepted last version of the article for publication. Figures have been produced by Pamir ATAGÜNDÜZ.
